# Intermediate phenotype between CMT2Z and DIGFAN associated with a novel *MORC2* variant: a case report

**DOI:** 10.1038/s41439-024-00287-8

**Published:** 2024-08-15

**Authors:** Kenta Hanada, Yusuke Osaki, Ryosuke Miyamoto, Kohei Muto, Shotaro Haji, Keyoumu Nazere, Yuki Kuwano, Hiroyuki Morino, Yoshiteru Azuma, Satoko Miyatake, Naomichi Matsumoto, Yuishin Izumi

**Affiliations:** 1 Department of Neurology, Tokushima University Graduate School of Biomedical Sciences, Tokushima, Japan; 2 Naka Municipal Kaminaka Hospital, Naka, Japan; 3 Department of Medical Genetics, Tokushima University Graduate School of Biomedical Sciences, Tokushima, Japan; 4 Department of Human Genetics, Yokohama City University Graduate School of Medicine, Yokohama, Japan

**Keywords:** Rare variants, Structural variation

## Abstract

Charcot-Marie-Tooth disease type 2Z is caused by *MORC2* mutations and presents with axonal neuropathy. *MORC2* mutations can also manifest as developmental delay, impaired growth, dysmorphic facies, and axonal neuropathy (DIGFAN). We report a patient exhibiting an intermediate phenotype between these diseases associated with a novel *MORC2* variant. A literature review revealed that the genotype‒phenotype correlation in *MORC2*-related disorders is complex and that the same mutation can cause a variety of phenotypes.

Charcot-Marie-Tooth disease (CMT) is a prevalent hereditary peripheral neuropathy that can be divided into axonal (median motor nerve conduction velocity >38 m/s) and demyelinating (median motor nerve conduction velocity <38 m/s) types^1^. To date, more than 120 genes have been identified as being responsible for CMT^2^, including at least 41 genes responsible for CMT2^3^.

CMT2Z, caused by mutations in the microrchidia CW-type zinc finger 2 (*MORC2*) gene, was first reported in 2016, and more than 30 families with this condition have been reported^4,5^. CMT2Z is an axonal peripheral neuropathy characterized by distal lower limb muscle weakness and sensory impairment^4^. Asymmetric axonal motor neuropathy is a characteristic clinical feature of CMT2Z.

Here, we report a patient with a novel variant in *MORC2* who presented with a distinctive facial appearance, mild intellectual disability, and asymmetric muscle weakness in the arm muscles.

A 56-year-old man presented with asymmetric weakness of the proximal arm muscles and distal muscles of all limbs. He had type 2 diabetes with nephropathy and retinopathy. A detailed interview revealed that he has had mild difficulty understanding complex stories and controlling his behavior since childhood. His highest level of education was a high school diploma, and he was single. His brother had difficulty running in childhood, and his parents did not exhibit any neurological symptoms. His brother and parents had already died at the time of this examination. The patient joined a track team in junior high school but had trouble running after graduation. He worked as a construction worker when he was in his thirties but could not wave a flag due to muscle weakness in his upper arm. He began to experience occasional cramps in his upper arm. When he was in his forties, he could not run due to weakness in his legs. At age 55, he had difficulty holding chopsticks due to weakness in his fingers. On neurologic examination at age 56, the distal muscles of his limbs exhibited atrophy and decreased strength, which was predominant in the lower limbs. He also exhibited patchy weakness in the arm muscles. The strength of the proximal lower limb muscles was normal. The left biceps and triceps tendon reflexes were decreased, and the bilateral Achilles tendon reflexes were absent. Superficial sensation was normal, but vibration sensation in the lower limbs was mildly decreased. There were no autonomic symptoms. The patient had an elongated face with a narrow jaw and a thin upper lip (Fig. 1a). The Mini-Mental State Examination score was 22/30, and the Frontal Assessment Battery score was 7/18. Brain magnetic resonance imaging (MRI) at age 58 revealed slight atrophy of the cerebral cortices and hippocampus, enlarged lateral ventricles, and high signals in the right corona radiata and deep cerebral white matter on fluid-attenuated inversion recovery (FLAIR) images (Fig. 1b). There were no abnormal signals in the corpus callosum. At age 56, nerve conduction studies revealed decreased compound muscle action potential amplitudes in the left median, ulnar, and peroneal nerves. Additionally, the ulnar nerve showed an asymmetric reduction in compound muscle action potential amplitudes. There was no evidence of demyelinating neuropathy. The sensory nerve action potential amplitude was not evoked in the sural nerve. Compared with those at age 50, the compound muscle action potential amplitudes were decreased (Table 1). The patient was clinically diagnosed with CMT2, and genetic analysis was performed. Exome sequencing revealed a novel heterozygous variant in *MORC2* (NM_001303256.3: c.1271 C > A, p.Thr424Lys), which was located in a known hotspot (Figs. 1c and 2). According to the American College of Medical Genetics and Genomics and the Association of Molecular Pathology guidelines, the variant was classified as likely pathogenic, fulfilling the PM2, PM5, PP2, and PP3 criteria. The variant has been registered in ClinVar as likely pathogenic. We obtained appropriate written consent from the patient for genetic testing and the publication of this article. Genetic analysis of his family could not be performed because they were already deceased.

**Fig. 1:**
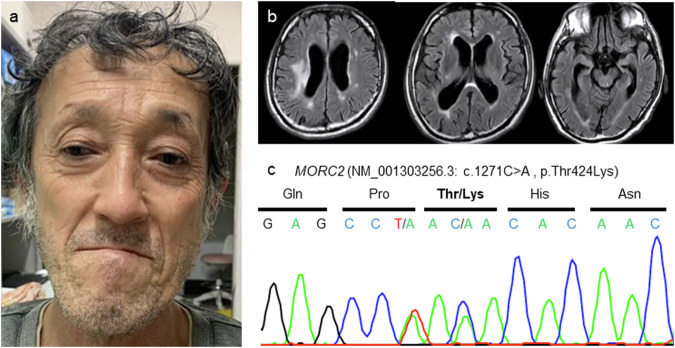
The patient's facial features, brain MR image, and sequencing analysis of *MORC2.* **a** Facial features of the patient. The patient presented with a long face, narrow jaw, and thin upper lip. **b** Brain MR image of the patient. MRI revealed slightly atrophied cortices and hippocampi, enlarged lateral ventricles, and high signals in the right corona radiata and deep cerebral white matter on FLAIR imaging. **c** Sequencing analysis of *MORC2* in the patient. Exome sequencing revealed a novel heterozygous variant in *MORC2* (NM_001303256.3: c.1271 C > A, p.Thr424Lys).

**Table 1:** Electrophysiological data.

	At age 50		At age 56	
	Nerve	Amplitude	Amplitude	
		Right	Right	Left
Motor	Median	1.7 mV	0.4 mV	0.6 mV
	Ulnar	7.3 mV	3.8 mV	0.9 mV
	Tibial	12 mV	–	–
	Peroneal	0.2 mV	No response	0.2 mV
Sensory	Median	6.6 μV	9.3 μV	2 μV
	Ulnar	13 μV	3.9 μV	3 μV
	Radial	–	9.1 μV	11 μV
	Sural	1.6 μV	–	No response

–: no record.

**Fig. 2:**
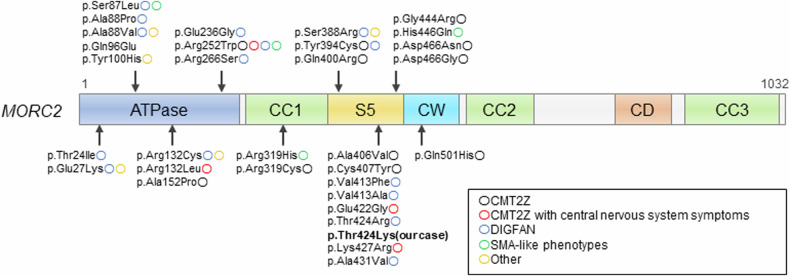
*MORC2* protein structure and reported variants and phenotypes. CC coiled-coil insertion domain, S5 ribosomal protein S5 domain, CW CW-type finger domain, CD chromo-like domain.

CMT2Z is caused by mutations in *MORC2*, which encodes an ATPase involved in epigenetic gene silencing^4,6^. In a mouse model, *Morc2* was found to be expressed in both the axons and Schwann cells of peripheral nerves, and *Morc2* mutations caused axonal degeneration^4^. CMT2Z is an axonal motor and sensory neuropathy that typically manifests in childhood or early adulthood^4^. Notably, asymmetric axonal motor neuropathy has been documented in several CMT2Z patients^4^. The main pathological feature was a multifocal pattern of myelinated fiber loss, which was consistent with asymmetric and random muscle weakness^4^.

Subsequent reports have revealed that mutations in *MORC2* can present a wide range of phenotypes^7^; heterozygous mutations in *MORC2* can cause spinal muscular atrophy-like (SMA-like) phenotypes and developmental delay, impaired growth, dysmorphic facies, and axonal neuropathy (DIGFAN) phenotypes, both of which develop earlier than CMT2Z^7^. The genotype‒phenotype correlation is not always apparent, but DIGFAN and SMA-like phenotypes are often caused by mutations that result in significant changes in biochemical properties; the Ser87Leu and Thr424Arg mutations may cause congenital or infantile onset of neuropathies by constitutively inducing N-terminal dimerization and increasing ATPase activity 3-fold, respectively^8^.

Our patient presented with muscle weakness in the lower limbs as well as asymmetric weakness in the upper limbs, which is a unique feature associated with CMT2Z. However, the distinctive facial features, including an elongated face with a narrow jaw and a thin upper lip, were reminiscent of those observed in DIGFAN. He also had a covert intellectual disability. MRI revealed cortical atrophy with ventriculomegaly and high white matter signals on FLAIR images, reminiscent of findings previously reported in DIGFAN patients. However, the patient did not have the severe developmental delay or impaired growth typical of DIGFAN. Thus, our patient’s phenotype was considered intermediate between those of CMT2Z and DIGFAN patients.

We performed a literature review on the genotype‒phenotype correlation of patients with *MORC2* mutations and found that even the same mutation may cause a variety of phenotypes (Supplementary Table 1); for example, the Arg252Trp mutation may cause CMT2Z, SMA-like phenotypes, CMT2Z with central nervous system symptoms or DIGFAN^4,9–14^. Interestingly, Thr424Arg, a different mutation of the same amino acid as in the present case, has been reported to cause typical DIGFAN^15,16^. Further research is needed to clarify whether the intermediate phenotype in our patient was due to the specific property of Thr424 or to another genetic or nongenetic factor. Finally, this case illustrates the broader phenotypic variation of *MORC2*-related disorders and further reveals a complex genotype‒phenotype correlation associated with *MORC2* mutation.

## HGV Database

The relevant data from this Data Report are hosted at the Human Genome Variation Database at: 10.6084/m9.figshare.hgv.3418, 10.6084/m9.figshare.hgv.3421.

## Supplementary information

Supplemental Table 1
