# Supplementary material for: Intermediate phenotype between CMT2Z and DIGFAN associated with a novel MORC2 variant: a case report
Source: Hum Genome Var. 2024 Aug 15;11:29. doi: 10.1038/s41439-024-00287-8 (PMC11324651; doi:10.1038/s41439-024-00287-8)
Supplement: Supplementary file 1 — Supplemental Table 1 [file 41439_2024_287_MOESM1_ESM.docx]

Supplemental Table 1. Genotype-phenotype correlation in patients with *MORC2* mutations.

| variant | symptoms | | | | | |  | phenotype | | | |  | references |
| --- | --- | --- | --- | --- | --- | --- | --- | --- | --- | --- | --- | --- | --- |
|  | intellectual disability | impaired growth | dysmorphic facies | central nervous system symptoms | proximal muscle weakness | neuropathy |  | CMT2Z | CMT2Z with central nervous system symptoms | DIGFAN | SMA-like phenotypes | Other |  |
| p.Thr24Ile | ✓ | ✓ | ✓ | ✓ |  |  |  |  |  | ✓ |  |  | Guillen Sacoto MJ et al, 2020 |
| p.Glu27Lys | ✓ | ✓ | ✓ | ✓ |  | N/A |  |  |  | ✓ |  |  | Guillen Sacoto MJ et al, 2020 |
|  | ✓ | ✓ |  | ✓ |  | ✓ |  |  |  | ✓ |  |  | Guillen Sacoto MJ et al, 2020 |
|  | ✓ | ✓ | ✓ |  |  |  |  |  |  |  |  | ✓ | Kistol D et al, 2023 |
|  | ✓ | ✓ |  | ✓ | ✓ |  |  |  |  | ✓ |  |  | Stafki SA et al, 2023 |
| p.Ser87Leu |  |  |  |  | ✓ | ✓ |  |  |  |  | ✓ |  | Sevilla T et al, 2016 |
|  | ✓ | ✓ | ✓ |  | ✓ | ✓ |  |  |  | ✓ |  |  | Hyun YS et al, 2016 |
|  | ✓ | ✓ |  |  | ✓ | ✓ |  |  |  | ✓ |  |  | Hyun YS et al, 2016 |
|  |  | ✓ |  |  | ✓ | ✓ |  |  |  |  | ✓ |  | Stettner G et al, 2019 |
|  | ✓ | ✓ | ✓ |  |  | ✓ |  |  |  | ✓ |  |  | Guillen Sacoto MJ et al, 2020 |
|  | ✓ | ✓ |  |  | ✓ | ✓ |  |  |  | ✓ |  |  | Guillen Sacoto MJ et al, 2020 |
|  |  | ✓ |  |  | ✓ | ✓ |  |  |  |  | ✓ |  | Yamamoto et al, 2021 |
|  | ✓ | ✓ |  | ✓ | ✓ | ✓ |  |  |  | ✓ |  |  | Duan X et al, 2021 |
|  |  | ✓ |  |  | ✓ | ✓ |  |  |  |  | ✓ |  | Sivera R et al, 2021 |
|  | ✓ | ✓ |  |  | ✓ | ✓ |  |  |  | ✓ |  |  | Stafki SA et al, 2023 |
| p.Ala88Pro | ✓ | ✓ | ✓ | ✓ |  |  |  |  |  | ✓ |  |  | Arbide D et al, 2024 |
| p.Ala88Val | ✓ | ✓ | ✓ | ✓ |  | N/A |  |  |  | ✓ |  |  | Guillen Sacoto MJ et al, 2020 |
|  | ✓ | ✓ | ✓ | N/A | ✓ | N/A |  |  |  |  |  | ✓ | Stafki SA et al, 2023 |
| p.Gln96Glu | N/A | N/A | N/A | N/A | N/A | N/A |  |  |  |  |  |  | Albulym OM et al, 2016 |
| p.Tyr100His | ✓ | ✓ | ✓ | ✓ | N/A | N/A |  |  |  |  |  | ✓ | Stafki SA et al, 2023 |
| p.Arg132Cys | ✓ | ✓ | ✓ |  |  | ✓ |  |  |  | ✓ |  |  | Guillen Sacoto MJ et al, 2020 |
|  |  | ✓ | ✓ | ✓ |  | ✓ |  |  |  |  |  | ✓ | Mirchi A et al, 2022 |
|  | ✓ | ✓ | ✓ | ✓ |  | ✓ |  |  |  |  |  | ✓ | Stafki SA et al, 2023 |
| p.Arg132Leu |  |  |  | ✓ | ✓ | ✓ |  |  | ✓ |  |  |  | Hyun YS et al, 2016 |
| p.Ala152Pro |  | ✓ |  |  |  | ✓ |  | ✓ |  |  |  |  | Sivera R et al, 2021 |
| p.Glu236Gly | ✓ |  |  |  | ✓ | ✓ |  |  |  | ✓ |  |  | Albulym OM et al, 2016 |
| p.Arg252Trp |  | ✓ |  | ✓ | ✓ | ✓ |  |  |  |  | ✓ |  | Sevilla T et al, 2016 |
|  |  | ✓ |  |  |  | ✓ |  | ✓ |  |  |  |  | Sevilla T et al, 2016 |
|  |  |  |  | ✓ | ✓ | ✓ |  |  | ✓ |  |  |  | Hyun YS et al, 2016 |
|  |  | ✓ |  |  | ✓ | ✓ |  |  |  |  | ✓ |  | Lassuthova P et al, 2016 |
|  |  | ✓ |  | ✓ | ✓ | ✓ |  |  | ✓ |  |  |  | Albulym OM et al, 2016 |
|  |  |  |  | ✓ | ✓ | ✓ |  |  | ✓ |  |  |  | Ando M et al, 2017 |
|  | ✓ | ✓ |  |  | ✓ | ✓ |  |  |  | ✓ |  |  | Ando M et al, 2017 |
|  |  | ✓ |  |  |  | ✓ |  |  | ✓ |  |  |  | Ando M et al, 2017 |
|  |  | ✓ |  |  | ✓ | ✓ |  | ✓ |  |  |  |  | Miressi F et al, 2020 |
|  | ✓ | ✓ |  | ✓ |  | ✓ |  |  |  | ✓ |  |  | Miressi F et al, 2020 |
|  |  | ✓ |  |  |  | ✓ |  | ✓ |  |  |  |  | Duan X et al, 2021 |
|  |  | ✓ |  |  |  | ✓ |  | ✓ |  |  |  |  | Sivera R et al, 2021 |
|  | ✓ |  |  |  |  | ✓ |  | ✓ |  |  |  |  | Nomura E et al, 2022 |
|  |  |  |  |  |  | ✓ |  | ✓ |  |  |  |  | Wang Y et al, 2022 |
|  |  |  |  |  |  | ✓ |  | ✓ |  |  |  |  | Mahungu AC et al, 2023 |
| p.Arg266Ser | ✓ | ✓ | ✓ | ✓ |  | N/A |  |  |  | ✓ |  |  | Guillen Sacoto MJ et al, 2020 |
| p.Arg319His | N/A | N/A | N/A | N/A | N/A | N/A |  |  |  |  | ✓ |  | Karakaya M et al, 2018 |
| p.Arg319Cys |  |  |  |  | ✓ | ✓ |  | ✓ |  |  |  |  | Sivera R et al, 2021 |
| p.Ser388Arg | ✓ | ✓ | ✓ | ✓ |  | N/A |  |  |  | ✓ |  |  | Guillen Sacoto MJ et al, 2020 |
|  |  | ✓ |  | ✓ |  |  |  |  |  |  |  | ✓ | Mekmangkonthong A et al, 2022 |
| p.Tyr394Cys |  | ✓ |  |  |  | ✓ |  | ✓ |  |  |  |  | Ando M et al, 2017 |
|  | ✓ | ✓ | ✓ |  |  | ✓ |  |  |  | ✓ |  |  | Guillen Sacoto MJ et al, 2020 |
|  | ✓ | ✓ |  |  |  | ✓ |  |  |  | ✓ |  |  | Guillen Sacoto MJ et al, 2020 |
|  |  | ✓ |  | ✓ |  | ✓ |  |  |  | ✓ |  |  | Frongia I et al, 2021 |
|  | ✓ | ✓ |  |  | ✓ | ✓ |  |  |  | ✓ |  |  | Sivera R et al, 2021 |
| p.Gln400Arg |  | ✓ |  |  | ✓ | ✓ |  | ✓ |  |  |  |  | Zhao X et al, 2016 |
|  |  |  |  |  | ✓ | ✓ |  | ✓ |  |  |  |  | Zhao X et al, 2016 |
|  |  | ✓ |  |  |  | ✓ |  | ✓ |  |  |  |  | Ando M et al, 2017 |
| p.Ala406Val |  | ✓ |  |  | ✓ | ✓ |  | ✓ |  |  |  |  | Sivera R et al, 2021 |
|  |  | ✓ |  |  | ✓ | ✓ |  | ✓ |  |  |  |  | Vujovic D et al, 2021 |
| p.Cys407Tyr |  | ✓ |  |  |  | ✓ |  | ✓ |  |  |  |  | Ando M et al, 2017 |
|  |  | ✓ |  |  |  | ✓ |  | ✓ |  |  |  |  | Duan X et al, 2021 |
| p.Val413Phe | ✓ | ✓ | ✓ |  |  | N/A |  |  |  | ✓ |  |  | Guillen Sacoto MJ et al, 2020 |
| p.Val413Ala | ✓ | ✓ |  | ✓ | ✓ |  |  |  |  | ✓ |  |  | Stafki SA et al, 2023 |
| p.Glu422Gly |  | ✓ |  | ✓ |  | ✓ |  |  | ✓ |  |  |  | Yang H et al, 2021 |
| p.Thr424Arg | ✓ | ✓ |  | ✓ | ✓ | ✓ |  |  |  | ✓ |  |  | Schottmann G et al, 2016 |
|  | ✓ | ✓ | ✓ | ✓ |  | ✓ |  |  |  | ✓ |  |  | Zanni G et al, 2017 |
| **p.Thr424Lys** | **✓** | **✓** | **✓** |  | **✓** | **✓** |  | **✓?** |  | **✓?** |  |  | **Our case** |
| p.Lys427Arg |  | ✓ |  | ✓ |  |  |  |  | ✓ |  |  |  | Stafki SA et al, 2023 |
| p.Ala431Val | ✓ | ✓ |  | ✓ | ✓ | ✓ |  |  |  | ✓ |  |  | Ando M et al, 2017 |
| p.Gly444Arg | N/A | N/A | N/A | N/A | N/A | N/A |  |  |  |  |  |  | Albulym OM et al, 2016 |
|  |  |  |  |  | ✓ | ✓ |  | ✓ |  |  |  |  | Jacquier et al, 2022 |
| p.His446Gln |  |  |  |  | ✓ | ✓ |  |  |  |  | ✓ |  | Jacquier et al, 2022 |
| p.Asp466Asn |  |  |  |  | ✓ | ✓ |  | ✓ |  |  |  |  | Zhao X et al, 2016 |
|  |  |  |  |  | ✓ | ✓ |  | ✓ |  |  |  |  | Semplicini C et al, 2017 |
| p.Asp466Gly |  |  |  |  | ✓ | ✓ |  | ✓ |  |  |  |  | Sivera R et al, 2021 |
|  |  |  |  |  |  | ✓ |  | ✓ |  |  |  |  | Duan X et al, 2021 |
| p.Gln501His |  |  |  |  |  | ✓ |  | ✓ |  |  |  |  | Gentile L et al, 2021 |

Abbreviations are as follows: N/A, not available; DIGFAN, developmental delay, impaired growth, dysmorphic facies, and axonal neuropathy; SMA-like, spinal muscular atrophy-like
